# Laryngeal Fracture after Blunt Cervical Trauma in Motorcycle Accident and Its Management

**DOI:** 10.1155/2017/9321975

**Published:** 2017-02-02

**Authors:** Nuno Ribeiro-Costa, Pedro Carneiro Sousa, Diogo Abreu Pereira, Paula Azevedo, Delfim Duarte

**Affiliations:** Hospital Pedro Hispano, Rua Dr. Eduardo Torres, Senhora da Hora, 4464-513 Matosinhos, Portugal

## Abstract

Laryngeal fracture is a rare traumatic injury, potentially fatal, with an estimated incidence of 1 in 30,000 patients admitted to severe trauma centers. Because of the rarity of this injury, physician may be not aware of its existence, leading to a late diagnosis of this entity. We report a case of a 59-year-old woman admitted to the emergency room after a motorcycle accident with cervical trauma. The patient presented with dysphonia, hemoptysis, cervical subcutaneous emphysema, and increasing respiratory distress that led to the intubation of the patient. CT-scan demonstrated displaced fracture of the cricoid and thyroid cartilage. The patient was submitted to tracheostomy and the fracture was surgically repaired. Tracheostomy was removed in third postoperative month. The patient presented a good recovery, reporting only hoarseness but without swallowing or breathing problems at 6-month follow-up.

## 1. Introduction

Laryngeal fracture is a rare traumatic injury, potentially fatal, with an estimated incidence of 1 in 30,000 patients admitted to severe trauma centers [1–4]. Factors such as mobility and elasticity of the larynx and its protection by the mandible and the sternum make it able to withstand severe trauma [2]. The rarity of this type of injury often leads to a delay in diagnosis which may contribute to airway patency problems, vocal production and swallowing [5]. Therefore it is important for clinicians who treat it to have a comprehensive understanding of its diagnosis and treatment in order to improve the patient outcome.

## 2. Case Presentation

**Figure 1:**
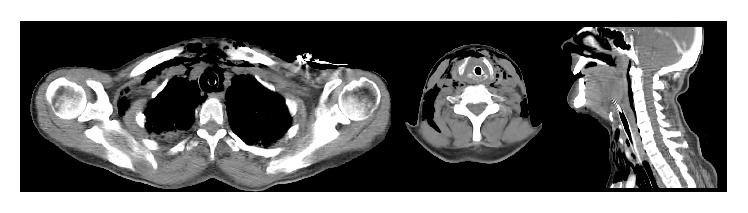
Neck and thorax CT-scan at admittance. It reveals anterior traumatic lesion of the larynx with severe emphysema of the cervical and supraclavicular area and displaced fracture of the thyroid cartilage. No laryngotracheal separation was observed.

A 59-year-old woman is admitted in the emergency room after a motorcycle accident with cervical trauma. The patient was conscious and oriented, presenting dysphonia, hemoptysis, and increasing respiratory distress that led to the intubation of the patient by an anesthetist in the emergency room. Her physical examination revealed a subcutaneous emphysema, edema, and tenderness in the cervical area, and other facial and extremity abrasions and ecchymosis. The cervical and thorax CT-scan demonstrated an anterior traumatic lesion of the larynx with severe emphysema of the cervical and supraclavicular area and fracture of the cricoid and thyroid cartilage (Figure 1).

**Figure 2:**
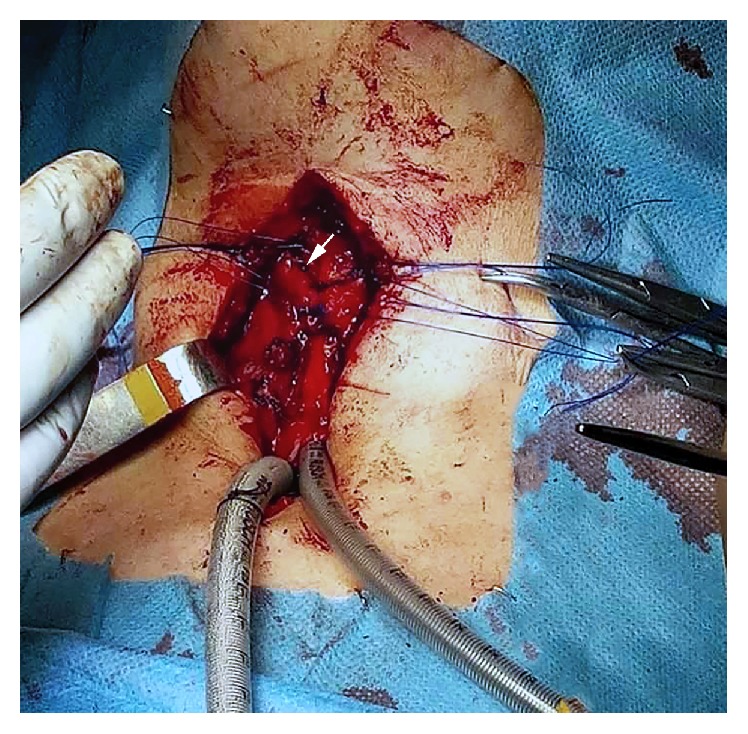
Intraoperative view of the laryngeal fracture (marked with a white arrow), with suture of the laryngeal framework.

After the patient stabilization, the patient was evaluated by otolaryngology and immediately admitted to the operating room. During surgery, a transversal fracture of thyroid and cricoid cartilage was found. The thyroid fracture was repaired and the cricoid cartilage was fixed to the thyroid cartilage with 3-0 prolene and tracheostomy was performed (Figure 2). The patency of the laryngeal lumen was maintained with an endotracheal tube, which was removed one week later.

The patient was initially admitted in intermediary care unit and fed through a nasogastric tube. The postoperative evaluation with flexible endoscopy revealed a bilateral paresis of the vocal cords and significant reduction of laryngeal sensibility and saliva aspiration. During her stay in the intermediary care unit the patient case was complicated with a pulmonary infection with multisensitive* Pseudomonas aeruginosa* which was successfully treated with piperacillin-tazobactam for 21 days. During this period the patient started sessions of speech therapy to help improved vocalization and deglutition. Before discharge the patient was submitted to surgical gastrostomy because of persistent food aspiration.

**Figure 3:**
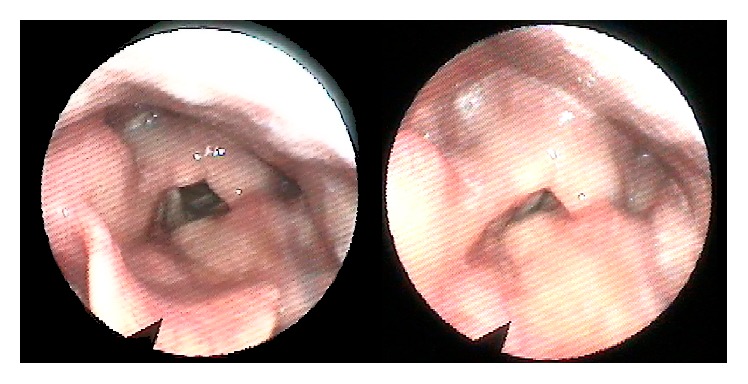
Flexible endoscopy demonstrating the postoperative view of the larynx.

At 3-month follow-up the flexible endoscopy revealed a normal mobilization of both vocal cords (Figure 3). However, during the swallowing evaluation, the patient still could not tolerate liquid food with occasional episodes of aspiration. At this time the tracheostomy was removed, and progressive oral feeding with creamy and solid food was started. Subsequently, at 6-month follow-up, the patient presented no evidence of secretions or food aspiration and the gastrostomy was removed. Although improved, her hoarseness still persists.

## 3. Discussion

Laryngeal fracture is an infrequent injury, most frequently resulting from anterior, blunt trauma to the neck from motor vehicles accidents, sports-related trauma, assault, or strangulation [4, 6]. Other causes include penetrating trauma due to gunshot or stab wounds to the neck [7]. Laryngeal trauma has a high mortality rate (17,9% to 40%), with many patients dying before reaching the emergency room because of severe airway injury or multiple organ injury [1, 4, 8].

In our case the patient presented with some of the classical, but not pathognomonic, symptoms such as hoarseness, anterior neck pain, progressive respiratory distress, hemoptysis, and cervical subcutaneous emphysema [5, 7]. However, some patients with laryngeal fracture may not present such symptoms, and a high level of suspicion is required of all anterior neck trauma [4, 6]. About 37% of the patients in reported series had delayed diagnosis [8]. CT-scan of the neck is considered the gold standard for diagnosing such injuries [3, 7, 9].

The primary objective in treating laryngeal trauma is maintaining the airway patency. Early airway management and aggressive physiologic compensation performed in the initial phase are an important determinant in the laryngeal trauma mortality [3, 10]. However, the most appropriate method for airway management is controversial. Both endotracheal intubation and tracheostomy have been recommended [2, 4, 6, 9]. Our patient was successfully intubated in the emergency room, but when severe trauma has been inflicted to the larynx, intubating can be extremely difficult because of distorted anatomy and poor visualization. The posterior conversion to tracheostomy after endotracheal intubation is also a good method, being recommended within 24 h because it decreases the length of hospitalization [3, 7, 8].

After safeguarding the airway, laryngeal treatment should be considered to improve the long-term voice and swallowing outcomes in these patients. Nondisplaced fractures can be managed nonoperatively, while surgical reduction of laryngeal framework should be performed in patients with displaced fractures [1, 7]. The timing of such repair is subject of debate but recent evidence suggests that early treatment within 48 hours resulted in a higher recovery rate than that of the delayed treatment group [6].
